# Supplementary material for: Human MicroRNAs Attenuate the Expression of Immediate Early Proteins and HCMV Replication during Lytic and Latent Infection in Connection with Enhancement of Phosphorylated RelA/p65 (Serine 536) That Binds to MIEP
Source: Int J Mol Sci. 2022 Mar 2;23(5):2769. doi: 10.3390/ijms23052769 (PMC8911160; doi:10.3390/ijms23052769)
Supplement: Supplementary file 1 [file ijms-23-02769-s001.zip › ijms-1595290-supplementary.pdf]

Supplementary Materials

A

0 h (Uninfected)

3'-UTR of *UL123*

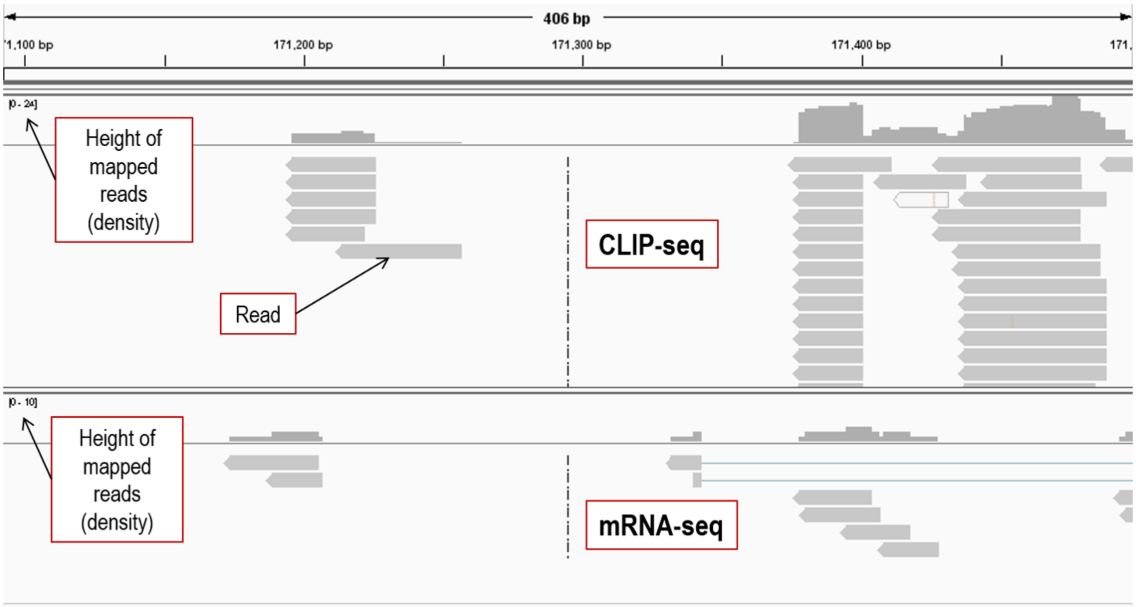

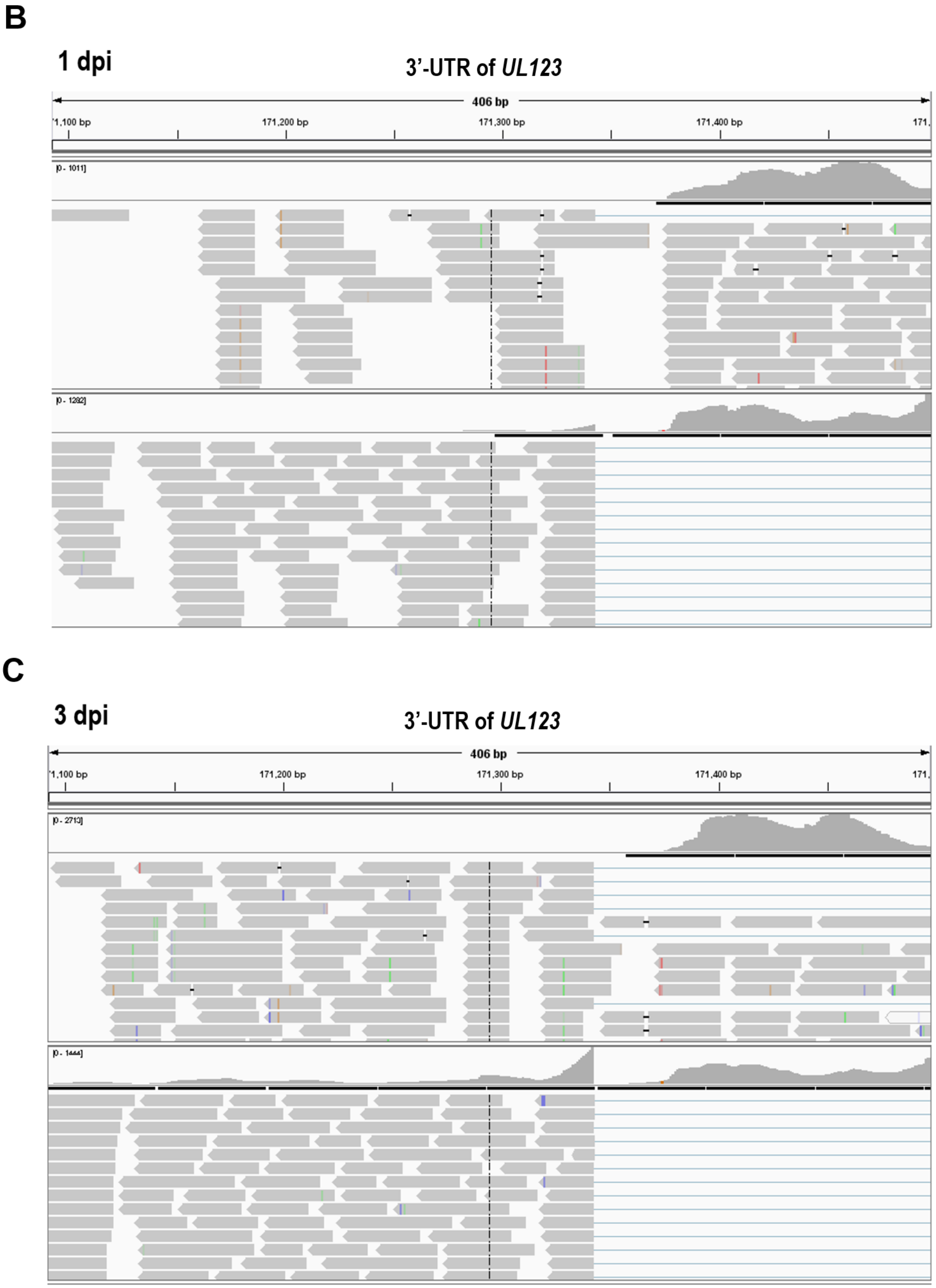

Supplemental Figure S1. AGO-CLIP-seq and mRNA-seq in the 3′-UTR of HCMV *UL123*.

Densities corresponding to the raw transcript reads in entire 3′-UTR of *UL123* (406 bp) from

AGO-CLIP-seq (upper panel) and mRNA-seq (lower panel) at 0 (A), 1 (B), and 3 dpi (C). We used high-throughput sequencing datasets from HFF-1 cells (GEO accession number: GSE63797) infected with the HCMV Towne strain (MOI of 1). Abbreviations: AGO, argonaute; CLIP, cross-linking immunoprecipitation; dpi, days post-infection; HCMV, human cytomegalovirus; HFF-1, human foreskin fibroblast-1; MOI, multiplicity of infection; seq, sequence; UL, unique long; UTR, untranslated region.

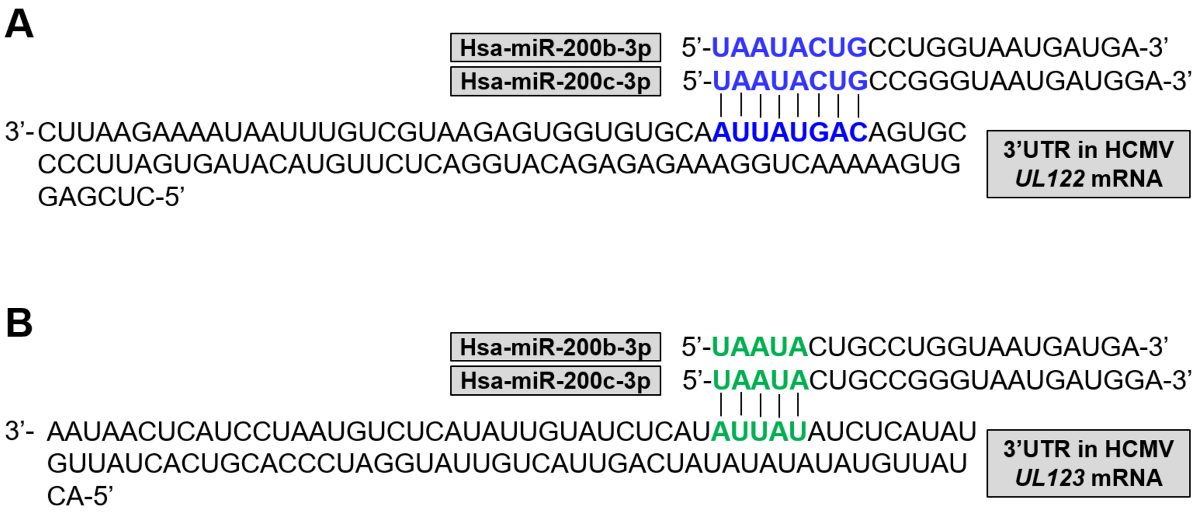

Supplemental Figure S2. Schema of the seed sequences of miR-200b-3p or miR-200c-3p that bind to the 3′-UTR of *UL122* or *UL123* mRNA. MiR-200b-3p and miR-200c-3p have identical seed sequences for *UL122*- (canonical, 8-mer) and *UL123*-3′-UTR (non-canonical, 5-mer). Bold font, binding sequences between miRNAs and mRNA. Abbreviations: HCMV, human cytomegalovirus; hsa, homo sapiens; miR, mature microRNA; UL, unique long; UTR, untranslated region.

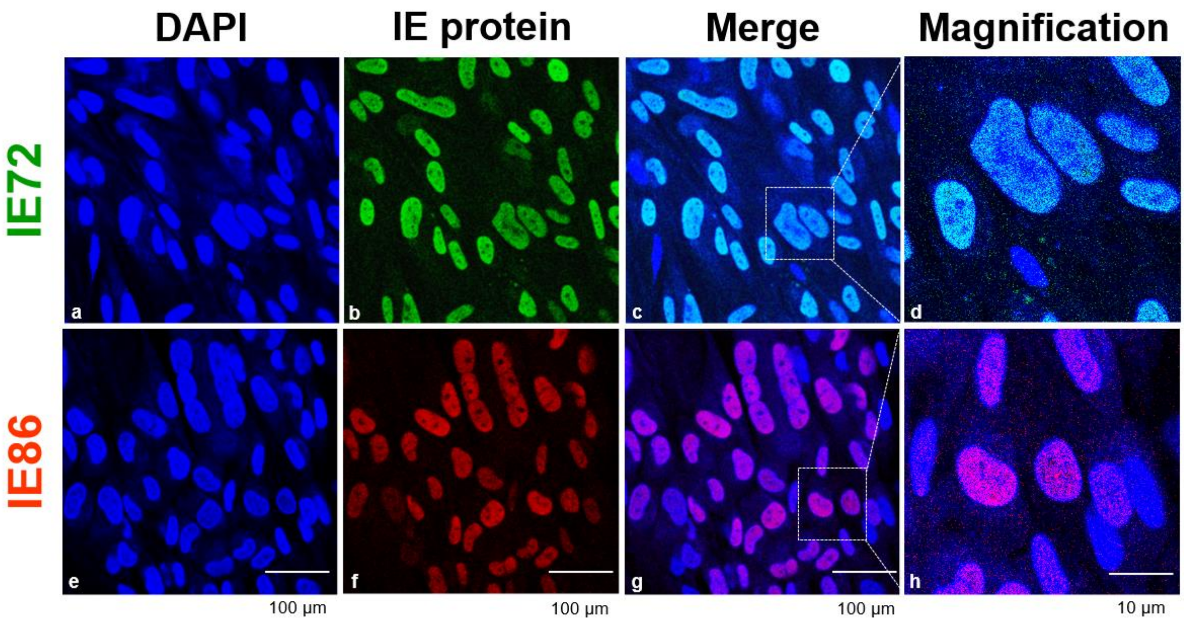

Supplemental Figure S3. Investigation of the localization and expression of HCMV IE72 and IE86 in HFF-1 cells infected with the HCMV Towne strain. HFF-1 cells (1×10^6/well) infected with the HCMV Towne strain at an MOI of 0.1 in a 6-well plate were stained with fluorescence-conjugated monoclonal antibodies to check for the expression of IE72 and IE86 and DAPI at 2 dpi. Fluorescence microscopy images show abundant nuclear expression of IE72 and IE86 at 2 dpi. Bars: a–c, and e–g, 100 µm; d and h, 10 µm. Abbreviations: DAPI, 4′,6-diamidino-2-phenylindole; dpi, days post infection; HCMV, human cytomegalovirus; HFF-1, human foreskin fibroblast-1; IE, immediate early protein; MOI, multiplicity of infection.

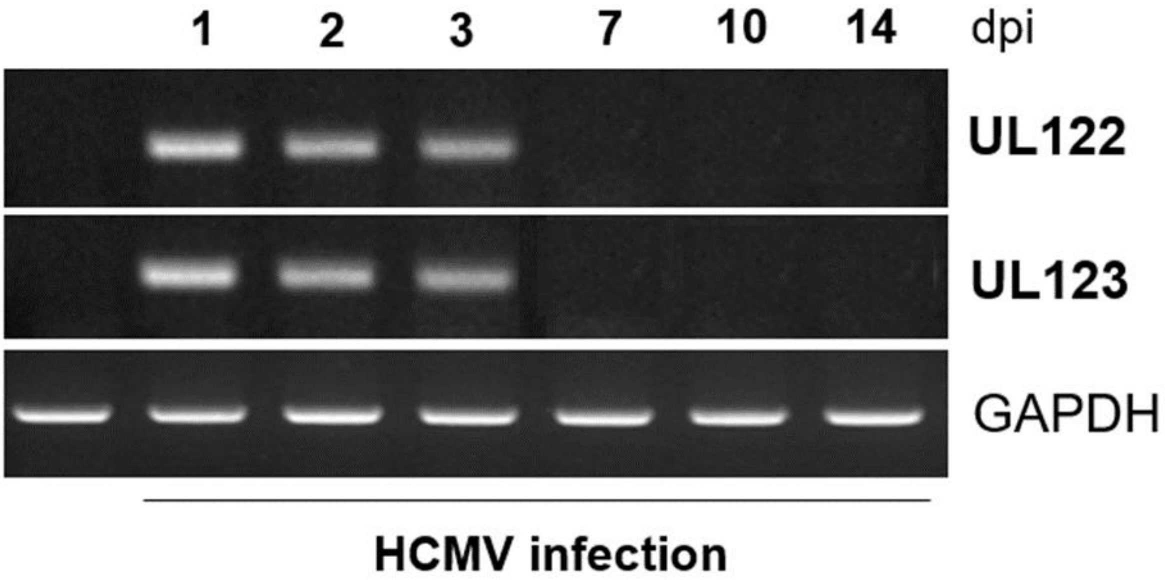

Supplemental Figure S4. Expression of HCMV *UL122* and *UL123* mRNA in latent-infected THP-1 cells. RT-PCR was used to measure the expression of HCMV *UL122* and *UL123* mRNA in THP-1 cells infected with the HCMV Toledo strain at MOI of 5 at indicated dpi. Expression of *UL122* and *UL123* mRNA at 1, 2, and 3 dpi, these molecules disappeared after 7 dpi during latent quiescence. Abbreviations: dpi, days post-infection; GAPDH, Glyceraldehyde 3-phosphate dehydrogenase; HCMV, human cytomegalovirus; MOI, multiplicity of infection; RT-PCR, reverse transcription-polymerase chain reaction; THP-1, human monocytic leukemia cell line; UL, unique long.

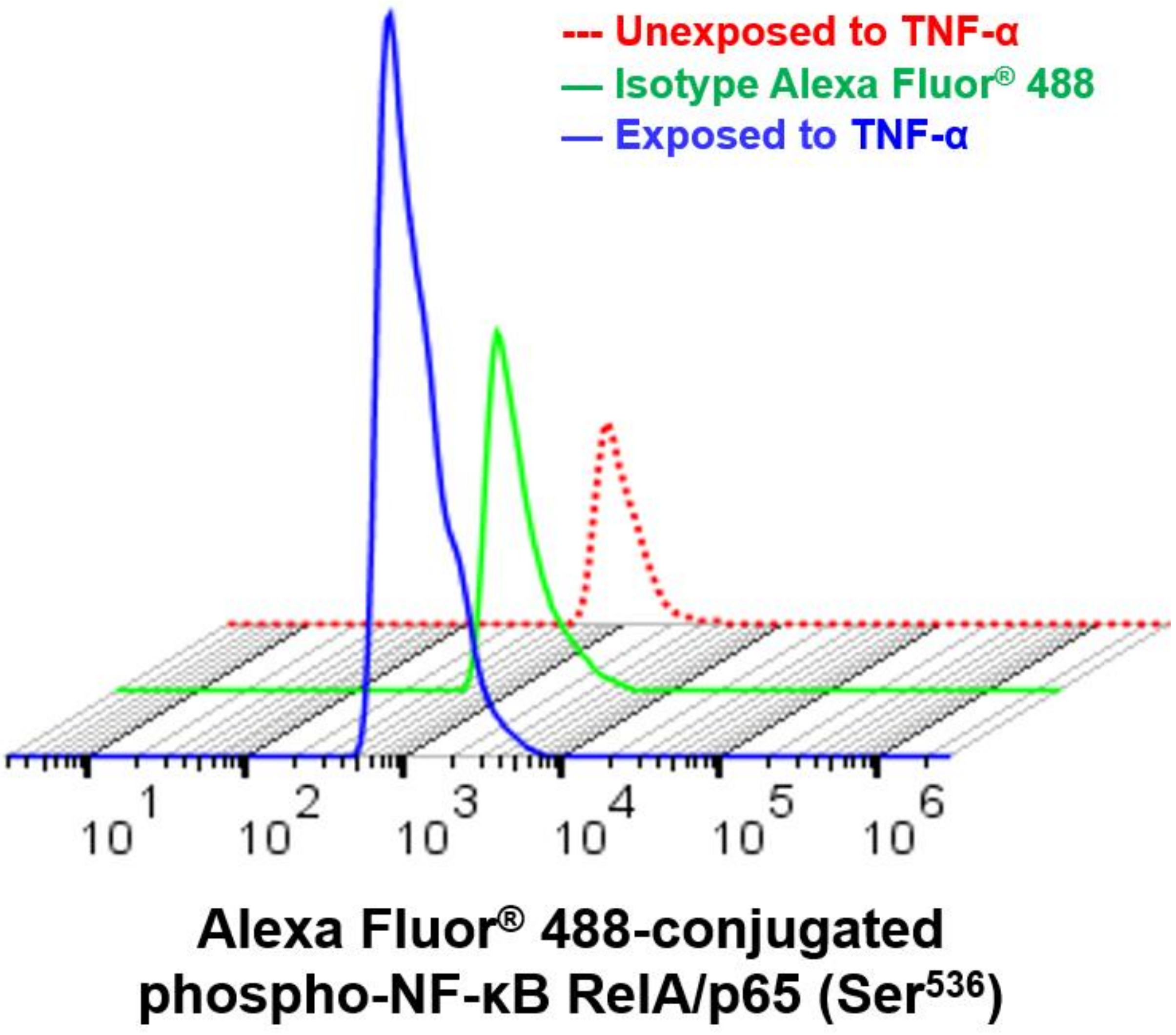

Supplemental Figure S5. Changes in the levels of p-RelA/p65(Ser^{536}) after TNF-α stimulation. We incubated HFF-1 cells (1 × 10^6/well) with 10 ng/mL recombinant TNF-α (human) for 0.5 h to confirm the expression of p-RelA/p65 (Ser^{536}) using flow cytometry. Expression of p-RelA/p65 (Ser^{536}) is significantly higher in cells incubated with—than without—TNF-α and anti-IgG-isotype control cells. Abbreviations: HFF-1, human foreskin fibroblast-1; IgG, immunoglobulin G; NF-κB, nuclear factor kappa-light-chain-enhancer of activated of B cells; p, phosphorylated; Ser, serine; TNF-α, tumor necrosis factor-α.

Supplementary Table S1. Reagents and resources used in the experiments

**Reagents or Resources**	**Source**	**Identifier**
**Cell Lines: Human**		Catalog No.
HFF-1	ATCC	SCRC-1041
U373MG	Korean Cell Line Bank	30017
THP-1	ATCC	TIB-202
**Chemicals and recombinant proteins**		Catalog No.
Recombinant human TNF-α	Cell Signaling	8902
Transfection Reagent		
Lipofectamine® RNAiMAX	Invitrogen	13778100
Lipofectamine® LTX & PLUS™	Invitrogen	A12621
**ChIP**		
Immunoprecipitation buffer	Abcam	ab206996
Chelex® 100	Bio-Rad	142-1253
Protein A-agarose beads (Sepharose®)	Abcam	ab193256
**Commercial Assays**		Catalog No.
mirVana™ PARIS Kit	Thermo Fisher Scientific	AM1556
TaqMan™ MicroRNA Reverse Transcription Kit	Applied Biosystems	4366596
TaqMan™ MicroRNA Assays for RT-PCR	Applied Biosystems	4427975
Hsa-miRNA-200b-3p		Assay ID: 002251
Hsa-miRNA-200c-3p		Assay ID: 002300

**Flow cytometry**		Catalog No.		
Cytofix/Cytoperm™ Fixation/Permeabilization buffer	BD Biosciences	554714		
Human Fcγ receptors blocking agent	BD Biosciences	564219		
**MicroRNAs**		Catalog No.		
*mir*Vana™ hsa-miRNA-mimics	Invitrogen	A25576		
598-3p		Assay ID: MC11417		
616-3p		Assay ID: MC12366		
662		Assay ID: MC11648		
892c-3p		Assay ID: MC24244		
1208		Assay ID: MC13539		
4676-3p		Assay ID: MC21749		
4716-5p		Assay ID: MC22455		
7159-3p		Assay ID: MC27720		
200b-3p		Assay ID: MC10492		
200c-3p		Assay ID: MC11714		
*mir*Vana™ hsa-miRNA-inhibitors	Invitrogen	A25576		
200b-3p		Assay ID: MH10492		
200c-3p		Assay ID: MH11714		
*mir*Vana miRNA mimic, Negative Control	Invitrogen	4464058		
**Monoclonal or polyclonal antibodies**		Catalog No.	RRID No.	Clone
**Immunoblotting**				
Mouse anti-GFP	Santa Cruz	sc-9996	AB_627695	B-2

Mouse anti-HCMV IE72	Santa Cruz	sc-69834	AB_1122105	6E1
Mouse anti-HCMV IE86	Santa Cruz	sc-69835	AB_1122106	12E2
Rabbit anti-NF-κB RelA/p65	Cell Signaling	8242	AB_10859369	D14E12
Rabbit anti-phospho-NF-κB RelA/p65 (Ser^{536})	Cell Signaling	3033	AB_331284	93H1
Rabbit anti-NF-κB p105/p50	Cell Signaling	13586	AB_2716496	D4P4D
Mouse anti-ß-actin	Sigma-Aldrich	A5441	AB_262011	AC-15
**Flow cytometry**				
Mouse anti-GFP-Alexa Fluor® 488 conjugate	Santa Cruz	sc-9996 AF488	AB_627695	B-2
Rabbit anti-NF-κB RelA/p65-Alexa Fluor® 488 conjugate	Cell Signaling	49445	AB_2799359	D14E12
Rabbit anti-phospho-NF-κB RelA/p65 (Ser^{536})-Alexa Fluor® 488 conjugate	Cell Signaling	4886	AB_390789	93H1
Rabbit anti-IgG-isotype control-Alexa Fluor® 488 conjugate	Cell Signaling	2975	AB_10699151	DA1E
FITC anti-human IL-1β	Invitrogen	11-7018-41	AB_1107016	CRM56
FITC anti-human IL-6	Invitrogen	11-7069-82	AB_465395	MQ2-13A5
FITC anti-human RNATES (CCL5)	Novus Biologicals	NB100-64262	AB_965231	VL1
**Immunocytochemistry**				
Mouse anti-HCMV IE72	Santa Cruz	sc-69834	AB_1122105	6E1
Mouse anti-HCMV IE86	Santa Cruz	sc-69835	AB_1122106	12E2
Rabbit anti-phospho-NF-κB RelA/p65 (Ser^{536})	Cell Signaling	3033	AB_331284	93H1
Goat anti-mouse IgG secondary Ab, FITC	Invitrogen	31630	AB_2534088	N/A
Goat anti-mouse IgG secondary Ab, Rhodamine	Invitrogen	31663	AB_2534091	N/A
**ChIP**				
Rabbit anti-NF-κB RelA/p65	Cell Signaling	8242	AB_10859369	D14E12
Mouse anti-phospho-NF-κB RelA/p65 (Ser^{536})	Santa Cruz	sc-136548	AB_2798185	E1Z1T

pEGFP-N1 plasmid vector	Takara Bio	6085-1
**Virus strains**		Catalog or Code No.
HCMV Towne strain	ATCC	VR-977
1st WHO International Standard for HCMV for NAT	NIBSC	09/162
Toledo strain	In this study	N/A

Aberrations: Ab, AB, antibody; ATCC, American Type Culture Collection; bp, base pair; ChIP, cross-linking chromatin immunoprecipitation; Fcγ, Fc-gamma receptors; FITC, fluorescein isothiocyanate; GFP, green fluorescence protein; hsa, homo sapiens; HCMV, human cytomegalovirus; HFF-1, human foreskin fibroblast-1; IE, immediate early; IgG, immunoglobulin G; miR, mature microRNA; N/A, not-applicable; NAT, Nucleic Acid Amplification Technique; No., number; NIBSC, the National Institute for Biological Standards and Control; pp, phosphoprotein; RRID, Research Resource Identifiers; RT-PCR, reverse transcription-polymerase chain reaction; Ser, serine; THP-1, TNF-α, Tumor necrosis factor-alpha; U373MG, human glioblastoma astrocytoma; UL, unique long; UTR, untranslated region; WHO, World Health Organization
